# Willingness to Participate in Health Information Networks with Diverse Data Use: Evaluating Public Perspectives

**DOI:** 10.5334/egems.288

**Published:** 2019-07-25

**Authors:** Jodyn Platt, Minakshi Raj, Ayşe G. Büyüktür, M. Grace Trinidad, Olufunmilayo Olopade, Mark S. Ackerman, Sharon Kardia

**Affiliations:** 1 University of Michigan Medical School, Department of Learning Health Sciences, US; 2 University of Michigan School of Public Health, Department of Health Management and Policy, US; 3 University of Michigan School of Information and Michigan Institute for Clinical and Health Research, US; 4 University of Chicago Medicine, US; 5 University of Michigan School of Information, College of Engineering, EECS, and Medical School, Department of Learning Health Systems, US; 6 University of Michigan School of Public Health, Department of Epidemiology, US

**Keywords:** health data networks, public opinion, trust, learning health systems

## Abstract

**Introduction:**

Health information generated by health care encounters, research enterprises, and public health is increasingly interoperable and shareable across uses and users. This paper examines the US public’s willingness to be a part of multi-user health information networks and identifies factors associated with that willingness.

**Methods:**

Using a probability-based sample (n = 890), we examined the univariable and multivariable relationships between willingness to participate in health information networks and demographic factors, trust, altruism, beliefs about the public’s ethical obligation to participate in research, privacy, medical deception, and policy and governance using linear regression modeling.

**Results:**

Willingness to be a part of a multi-user network that includes health care providers, mental health, social services, research, or quality improvement is low (26 percent–7.4 percent, depending on the user). Using stepwise regression, we identified a model that explained 42.6 percent of the variability in willingness to participate and included nine statistically significant factors associated with the outcome: Trust in the health system, confidence in policy, the belief that people have an obligation to participate in research, the belief that health researchers are accountable for conducting ethical research, the desire to give permission, education, concerns about insurance, privacy, and preference for notification.

**Discussion:**

Our results suggest willingness to be a part of multi-user data networks is low, but that attention to governance may increase willingness. Building trust to enable acceptance of multi-use data networks will require a commitment to aligning data access practices with the expectations of the people whose data is being used.

## Introduction

Health information generated by health care encounters, research enterprises, and public health is increasingly interoperable and shareable across uses and users. This paper examines the public’s willingness to be a part of large health data networks and identifies factors associated with this attitude.

Networks that support health data and information exchange are at the heart of health IT infrastructure development and are a necessary element of learning health care systems [1,2], accelerated quality improvement, and more efficient implementation and patient care. The scope of networked data ranges from patient-level clinical trial and research data [3,4] to genetic data [5,6,7,8] and electronic health record information [2, 9,10,11]. Network-to-network connectivity and the capacity to exchange health information, including PHI, is maturing. Commonwell, which enables querying of data for treatment and direct patient access, for example, has 17,609,390 enrolled (unique) individuals accessible in their network and is growing at a rate of ~900K monthly. eHealth Exchange supports 109 million patients and the Strategic HIE Collaborative currently has the capacity to exchange data from >195 million individuals from hospitals, health care providers and other participants serving nearly 75 percent of the US patient population. The Trusted Exchange Framework and Common Agreement proposed by the Office of the National Coordinator will provide additional support and incentivization to join these networks into nationwide networks of networks [12]. The Center for Medicaid and Medicare Services State Innovation Model initiatives are, in part, an investment in care coordination so that data sharing might be facilitated between social service agencies and health systems [13]. In the research domain, the NIH All of Us Initiative aims to connect genomic, health, social media, and user-generated data from >1,000,000 individuals to promote precision health and accelerate discovery from bench to bedside [14]. These large-scale initiatives will create an ecosystem that provides increased access to health information to support care coordination, quality improvement, and implementation research.

Studies investigating attitudes about access to genomic data, participation in population-based longitudinal cohort studies, and large population biobanks have identified concerns about privacy, discrimination, and objectionable research applications as particularly salient [15,16]. A recent review of studies on genomic data access found that altruism and contributing to the greater good as well as trust, transparency, and confidence in governance counterbalance these concerns [17,18]. Studies investigating public perspectives data access have found that many in fact support data accessibility in hopes learning about their own health [19] and for advancing research [7], but emphasize the need for disclosure about a study’s data-access intent during the informed consent process [20] and expect de-identification of data [21].

The public’s overall willingness to participate in a generalized case of networks that support health data exchange across diverse users, system-wide, from “bench to bedside” (i.e., inclusive of researchers, institutions, and providers), however, is less well-understood. Experience with a fragmented health care system and unreliable coverage, as an example, may leave the public skeptical about the capacity for health information users to realize the benefits they promise. Identifying factors associated with increased willingness to be a part of multi-user data networks highlights opportunities for outreach, policy intervention, or addressing system vulnerabilities.

In light of the current-day expansion of health information networks to include different types of data and an increasingly large number of individuals, we evaluated attitudes and beliefs that might be associated with willingness to be a part of networks that share health information with different types of users ranging from health care providers to social service agencies. We further examined what individual factors such as demographics as well as the experience and beliefs held about the health system that might directly inform one’s willingness to participate in a system that promotes broad access to health information. To this end we included questions regarding beliefs about medical conspiracies [22,23], beliefs about one’s obligation to participate in research [24,25,26], and general views of data access. Specifically, we hypothesized that beliefs about privacy, trust in the health system, having adequate insurance coverage, and attitudes about policy and governance might be associated with willingness to participate in data networks. Ethically, there is a strong and oft-cited argument for asserting an obligation for the public to participate in health information networks as learning health systems based on an assessment of the risks relative to considerable potential benefits to individuals and society; this argument led us to include questions about the relationship between this belief and willingness to participate in mutli-user data networks [27,28].

## Methods

We used GfK’s probability-based, nationally representative sample of U.S. adults (KnowledgePanel) to conduct an online survey in December 2016. One thousand fourteen (1,014) individuals participated and completed the survey (62.6 percent complete response rate). GfK calculated post-stratification weights corresponding to the U.S. Census demographic benchmarks for age, sex, household income, education, and race and ethnic background to reduce bias from random sampling error.

All survey participants viewed a 1.5-minute animated video describing the health system as the network of relationships among health care providers, departments of health, insurance systems, and researchers [29]. The video described how data sharing can happen in three ways: 1) patient-level information within a health data network for patient care, 2) patient-level information from the data network to external groups (e.g., other providers involved in patient care), and 3) summary information to be shared with external groups (e.g., aggregate data reporting to public health agencies or to researchers). To assess willingness to participate in health data networks, we asked whether participants were “comfortable having my electronic health information being a part of a network that includes” six different agencies or data networks: 1) other health care providers involved in my care, 2) all health care providers in my state, 3) social service agencies, 4) mental health services, 5) research networks, and 6) quality improvement networks. Participants rated their responses on a four-point “how true” scale ranging from “Not at all true [1]” to “Very true [4]”. We generated a measure of overall **willingness to participate in multi-user data networks** by summing the responses to questions about willingness to share data with each of the six potential participants in health data networks. The final dependent variable had a range of 6 to 24.

Hypothesized predictors of willingness to participate in data networks included demographic factors, as well as measures of trust: trust in the health system, trust in providers, and generalized trust; beliefs about privacy, beliefs about medical deception, having a positive view of data sharing, beliefs about policy and governance of data sharing, obligation to research, altruism, and concerns and confidence in participants’ health insurance coverage. Individual statements were evaluated on a four-point “how true” scale. Indices measuring trust in providers, concerns about privacy, belief in medical deception, altruism, and beliefs about policy and governance were calculated as the sum of responses for each question divided by the number of questions; indices have values of 1–4. Questions used in constructing these indices are listed in the Supplementary Material. Trust in the health system is a composite sum of four indices, calculated in the same way; this variable has a range of 4–16 [29]. Questions were derived and adapted from previously validated surveys [29,30] and by the research team.

**Figure 1:**
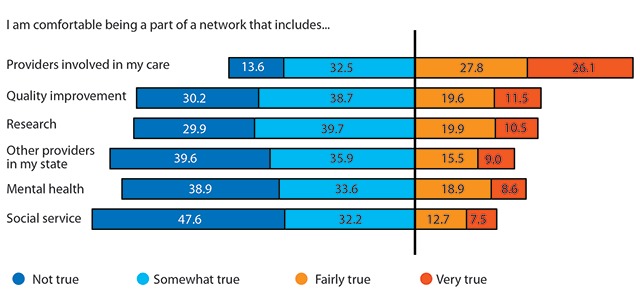
Percentage of respondents comfortable with being a part of data sharing networks.

For the present analysis, we used survey responses from 890 participants with complete data. To check that missingness was most likely random, we compared the demographics of participants with complete survey responses to those with missing data and did not find statistically significant differences across sex, age, race/ethnicity, education, income, or self-reported health. We first generated summary descriptive statistics of respondent characteristics (demographics), 13 predictor variables (See Table 1), and for the six questions evaluating willingness to participate in a data network (See Figure 1) that were used to create the dependent variable, willingness to participate in health data networks. We then conducted Weighted Ordinary Least Squares (OLS) regression analyses univariately. We evaluated the correlations (r) between predictor variables to evaluate potential collinearity and the direction and strength of the relationships (see Supplementary Material).

**Table 1:** Willingness to participate in data networks: Descriptive statistics and regression analyses.

	Characteristic	Frequency (n = 884) %/Mean (SD)	Univariable Weighted OLS b* (p value)	Multivariable Weighted Stepwise b* (p value) *R^2^ = 0.426*

**Outcome variable**	Willingness to participate in data networks (Range: 6–24)	12.6 (4.8)	N/A	N/A

**Demographics**	Age	52.9 (17.22)	0.001 (0.99)	
	Female	49.9%	–0.050 (0.23)	
	White Non-Hispanic	76.7%	0.001 (0.98)	
	Annual Income <$50,000	35.5%	0.065 (0.13)	
	Education			
	B.A. or higher	38.7%	REF	REF
	Some college	27.7%	0.122 (0.007)	0.009 (0.773)
	High school or less	33.6%	0.182 (<0.001)	–0.110 (0.003)
	Self reported health (Range: 1 = Excellent; 5 = Poor)	2.6 (0.94)	–0.076 (0.101)	

**Beliefs about the health system**	Medical Deception Index (Range: 1 = low; 4 = high)	1.75 (.65)	–0.178 (<0.001)	
	Health System Trust Index (Range 4 = low trust; 16 = high trust)	10.83 (2.08)	0.402 (<0.001)	0.094 (0.033)
	Provider Trust (Range: 1 = low trust; 4 = high trust)	2.83 (.64)	0.362 (<0.001)	
	Generalized Trust**	39.37%	0.357 (<0.001)	0.081 (0.030)
	Obligation to Participate in Research**	21.4%	0.335 (<0.001)	0.217 (<0.001)
	Altruism Index (Range: 1 = low altruism; 4 = high altruism)	2.77 (.65)	0.192 (<0.001)	

**Beliefs about insurance coverage and privacy**	Privacy Index (Range: 1 = low privacy concerns; 4 = high privacy concerns)	1.94 (.80)	–0.245 (<0.001)	–0.276 (0.002)
	Privacy*My health insurer could use information against me (interaction term)			0.298 (0.030)
	My health insurer could use my information against me	28.28%	–0.073 (0.115)	–0.063 (0.470)
	My health insurer could share my personal information with people who should not have it	33.94%	–0.100 (0.024)	
	I am worried about not being able to pay medical bills	25.23%	–0.085 (0.063)	
	I am confident my health insurance covers my medical needs	49.89%	0.264 (<0.001)	

**Beliefs about policy and governance**	Confidence in current governance (index) (Range: 1 = Low confidence; 4 = high confidence)	2.3 (0.74)	0.556 (<0.001)	0.393 (<0.001)
	I would like to give permission for health information to be shared in a network**	52.94%	0.162 (<0.001)	0.116 (0.001)
	I would like to be notified if my health information is shared**	64.14%	–0.036 (0.426)	–0.090 (0.012)

** Statement is Very or Fairly True. b*: Standardized regression coefficient.

Stepwise regression models were then employed using standard inclusion and exclusion selection methods (inclusion criteria (p < 0.05) and backward elimination using exclusion criteria (p ≥ 0.10)) to identify a parsimonious multivariable model of willingness to participate in data networks. Given the number of variables used in the multivariable regression models, the probability of making Type I errors (i.e., rejecting the null hypothesis when it is true leading to the identification of too many factors as “significant”) is higher than single multivariable modeling [31]. Bonferroni corrections were applied to stepwise regression models to generate more conservative estimates of statistical significance (p = α/*k*; α = 0.05 and *k* = number of parameters). Reported regression coefficients are standardized to allow comparison of the relative magnitude of the effect of each of the independent variables on willingness to participate in multi-user health networks.

We then examined the direction and strength of association of each variable in the stepwise model compared to its univariate regression relationship to identify potential model misspecifications due to interactions. Based on a suspected interaction between the privacy index and the belief that “my health insurer could use my information against me”, our final multivariable regression model includes this interaction term.

## Results

### Sample characteristics

Table 1 summarizes demographic characteristics of the sample. Our sample was nearly evenly split between men and women. Respondents ranged in age from 18–93; the median age was 52.9. Approximately three quarters were white, non-Hispanic (76.7 percent). Approximately one-third had a high school education or less (33.6 percent), 27.5 percent had some college and 38.7 percent had a bachelor’s degree or higher. Overall, 35.5 percent earned less than the median household income in the United States of $50,000K per year. On a scale of 1 to 5, with 1 being excellent and 5 being very poor, respondents rated their health as 2.6 on average, i.e., between very good and good.

We evaluated the attitudes and beliefs that we hypothesized might be associated with willingness to participate in data networks using variables capturing participants’ experience and views about their beliefs about the health system (belief in medical conspiracies, general view of data sharing, belief about one’s obligation to participate in research, insurance coverage, privacy, and trust in the health system, health care providers, and trust in general) and beliefs about policy and governance.

### Beliefs about the health system

We used three variables to measure trust in (a) the health system, (b) health care providers, and (c) in general. Average trust in health care providers was 2.8 (SD = 0.6), based on an index summarizing 5 questions with a scale of 1–4 (low to high trust). Trust in the health system, derived from the sum of 4 dimensions of trust (fidelity, integrity, competency, and global trust) with a range of 4–16 had a mean of 10.8 (SD = 2.1). Overall, 39.4 percent stated that it was fairly or very true that “most people can be trusted.” We also asked respondents how true it was that “People have an ethical obligation to participate in health research.” Approximately one in five felt this was true or very true (21.4 percent). The four-point altruism index had a mean of 2.8 and a standard deviation of 0.7.

We asked four questions about whether the health care system was intentionally deceptive (See Supplementary table). Belief in such practices is high. For example, nearly 60 percent believe that it is somewhat, fairly or very true that “the government does not tell the truth about the dangers of vaccines” and one quarter believe that “some medical research projects are secretly designed to expose minority groups.” Belief that “the health care system experiments on patients without them knowing about the experiments” was held by 42.8 percent of respondents and approximately three quarters (74.0 percent) say that “Health professionals don’t tell you everything you need to know about medicines.” Overall, 83 percent believe that it is at least somewhat true that the health system engages in one or more of these practices. The mean of the medical deception index was 1.8, with a standard deviation of 0.7.

### Beliefs about insurance coverage and privacy

Approximately 28 percent of respondents expressed a belief that their health insurer could use private information against them. One-third (33.9 percent) believed their insurer could share personal information with people who “have no business knowing it.” In terms of confidence in their health insurance coverage, about one in four (25.2 percent) were worried about not being able to pay medical bills. Only half are confident that their insurance covers their medical needs. We asked five questions about peoples’ concerns about privacy in the health system (See Supplementary Material). The average of the privacy index, which had a scale of 1–4, with 1 being low privacy concerns and 4 being high privacy concerns, had an average value of 1.9 (SD = 0.8).

### Beliefs about policy and governance

We asked seven questions evaluating attitudes and beliefs about preferences for policies and governance of health information. Three questions examined general satisfaction with the regulatory environment; Approximately one-third felt access the electronic health information is adequately regulated (31.4 percent); 35.9 percent felt electronic health information is sufficiently protected by law and regulation, and 41.9 percent stated confidence in the standards for confidentiality of personal health information. We further asked about the accountability of health researcher and physicians in conducting ethical research; 44.6 percent and 48.0 percent stated that it was fairly or very true that health researchers and physicians are sufficiently accountable, respectively. These five questions were combined into a single index (Chronbach’s alpha = 0.89) that has a range of 1–4; mean 2.3, and standard deviation 0.74).

An additional two questions evaluated preferences for notification and consent (permission). Most stated a preference for notification (64.1 percent), while respondents varied widely with respect to a preference for giving permission for health information to be shared in a network: 26.7 percent “Very true”, 26.2 percent “Fairly true”, 25.8 percent “Somewhat true”, and 21.3 percent “Not at all true.”

### Willingness to participate in data networks (dependent variable)

Willingness to participate in health data networks that include health care providers involved in one’s care, in one’s state, mental health services, social services, research networks, or quality improvement networks is low (See Figure 1). Willingness to have one’s health information in a network of personal providers is only 26 percent. The number declines by more than half for generalized networks of providers in one’s state (9.0 percent), social service agencies (7.4 percent), mental health services (8.5 percent). Research networks (10.5 percent) and quality improvement networks (11.5 percent) were not significantly different (p < 0.001, analysis not shown). Overall willingness to participate, measured as the sum of responses to each of the six potential participants in data networks, had a mean of 12.6 and a standard deviation of 4.8 (Range: 6–24).

### Predictors of willingness to participate in data networks: Regression models

We evaluated the **univariable relationship** between willingness to participate in data networks and the variables described in Table 1. Of these, we found that education and income were positively associated with willingness to participate, i.e., those with some college (b* = 0.12, p = 0.007) or a BA or higher (b* = 0.18, p < 0.001) were more willing to participate than those with a high school degree or less. Those who earned less than median income ($50,000 per year) were less likely to be willing to participate (b* = –0.09, p = 0.027). Sex, age, race, income, and self-reported health were not statistically associated with willingness to participate.

Those who said they were confident that their health insurance covered their medical needs were more likely to be willing to participate in data networks (b* = 0.26, p < 0.001). Concerns about privacy (b* = –0.25, p < 0.001) and greater belief in medical deception (b* = –0.18), p < 0.001) were negatively associated with the outcome of interest. All three measures of trust were positively associated with willingness to participate (p < 0.001), as was the view that people have an obligation to participate in research (b* = 0.34). Concern about not being able to pay for medical bills was not a significant predictor.

The index of confidence in current governance of health information was univariably associated with willingness to participate in data networks. Greater confidence in governance was associated with greater willingness (b* = 0.56, p < 0.001). Notably, those who stated a preference for giving permission for health information to be shared in a network also expressed greater willingness to participate in data networks (b* = 0.15, p < 0.001). Preference for notification was not a statistically significant factor (b* = –0.04, p = 0.43).

### Stepwise regression model

The results of the stepwise regression model are summarized in Table 1. Willingness to participate in a health information network was significantly associated with nine factors. Confidence in current governance was most strongly associated with willingness to participate (b* = 0.39, p < 0.001). The belief that people have an obligation to participate in research was also strongly positively associated factor (b* = 0.22, p < 0.001) as well as the desire to give permission for heath information to be shared in a network (b* = 0.12, p = 0.001), trust in the health system (b* = 0.09, p = 0.03), and generalized trust (b* = 0.08, p = 0.03).

Factors that were negatively associated with willingness to participate in multi-user health data networks included having a high school education or less (b* = –0.11, p = 0.003; Ref = college or greater), and privacy as measured by the privacy index (b* = –0.28, p = 0.002). The statistically significant interaction of privacy with fear of harm, i.e., the belief that “my health insurer could use my information against me” (b* = 0.30, p = 0.030) suggests that as privacy concerns and fear of harm increase, concerns about participating in data networks are magnified beyond the additive contribution of both concerns. Preference for notification was also negatively associated with willingness to participate such that those who were more willing to participate in information networks were less likely to state a preference for notification (b* = –0.09, p = 0.012).

## Discussion

Our results suggest willingness to participate in multi-user health information networks is low, but that attention to governance and trust building are associated with increased willingness. Our study found greater willingness was positively associated with confidence in current governance such as accountability of researchers and adequate regulatory safeguards. Governance should thus prioritize transparency about safeguards and accountability [10]. Just as confidence in policy was positively associated with willingness, participants with concerns about privacy or deception were less willing to participate. Moreover, the interaction of privacy with a fear of harm associated with greater access to data by a diverse set of users suggests that there is a need for having a robust policy and governance for data access that is known and accountable to the public. Concerns about privacy may be allayed if policies and practice come with an assurance that people will not be harmed by data networking. Efforts should be made to ensure that information is not used to discriminate or harm. Current legal protections should be made clear to the public, and further engagement should identify additional opportunities for regulatory modernization.

Participants who wanted to give permission for data sharing were more willing to be in multi-user networks. This suggests that even for trusting consumers, options for consent, dialogue, and innovative engagement in data governance is preferable to treating them as bystanders. There was considerable variability in the preference for giving permission and yet, the preference for giving permission extended to each type of data network use and user in the summary measure we used (p < 0.05), except for providers involved in direct patient care (analysis not shown). Thus consent and notification that are managed according to personal (consumer) preferences might be preferable to current models that provides waivers of consent or requires entire consumer groups to opt-in or opt-out.

With respect to trust, we found that the public is not particularly trusting. Less than half of respondents reported that in general, most people can be trusted; approximately 55 percent of respondents stated that they have trust in the health system, generally; trust in providers was higher at ~86 percent. The high levels of skepticism that we found in our study are consistent with prior research describing the public’s fear of misuse or intentional abuse of their data [32,33,34]. Prior studies have found that support for data sharing increases when trust in the individuals or organizations conducting the research increases, and further, when participants perceive potential benefits from the research being conducted [35]. Further research that empirically investigates trust building amongst stakeholders – including the public – in networked health information is needed to ensure willingness to participate in health information networks and the success of learning health systems and large-scale implementation or precision health initiatives.

Having at least a college education as well as belief in an ethical obligation to participate in research and having generally positive views of data sharing were all associated with an increased willingness to participate in multi-user health networks. These findings suggest we may be formulating policy and practice around data access according to proximity principles and homophily [36,37], thus creating systems that do not reflect the values and needs of the people it intends to serve. According to theories of proximity and homophily, individuals and communities tend to act in accordance with those individuals and communities that are most like them. Applied in the context of health information exchange and policies for expansion, proximity principles would suggest that those who make decisions about data access are also those who are most likely to favor it for the purposes we studied (care, quality improvement, research, social services), and thus make policy to maximize data exchange. Our findings suggest that the consumer community may not share this level of comfort or support for policies that facilitate the expansion of health data users. This is an opportunity for a reflexive form of governance that is aware of whether and how they are creating policy built on institutional or on societal values. Notably, greater willingness to participate in health data networks was negatively associated with a desire to be notified about data use, suggesting that the more one is comfortable, the less one feels a need for active engagement with how health information is used.

Two potential options for future governance of data sharing networks are to reduce data access to reflect the general public willingness to participate in data networks or, alternatively, to address concerns to ultimately improve the general public’s willingness to participate in data networks. The former faces considerable barriers given the current momentum toward expansion of data networks and current policy and practice that facilitate the exchange of data without consent, as is the case for quality improvement. The latter will require investment from institutions that participate in health information sharing networks, but engagement and dialogue with the public can be pursued in parallel with existing efforts. The Draft Trusted Exchange Framework and Common Agreement [12], which includes provisions for consent and withdrawal of consent could go further to elaborate on best practices for building and maintaining public trust.

For example, beyond explaining use of data to the public, notifying participants in data networks prior to using their data could be more broadly pursued. It is important in this regard for providers, researchers, and the health system at large to respect that participants still have an affinity with their data and may not simply become disconnected following collection. Furthermore, the differentiations among data use that guide permissions, i.e., that the same data may be used in quality improvement or for research, may be nuances that are opaque to participants. Informed participants must not only be equipped with information about their participation and their data, but further, should have the option to decline the health system’s use of their data for a broad set of uses.

Improving processes of building trust, increasing transparency, and ensuring fair usage of data requires governance that integrates stakeholder engagement. Based on the findings presented here, stakeholders who are immersed in current health data governance and use – from collection and aggregation to dissemination and policymaking – need to recognize and consider the perspectives of the public as patients or participants who are contemplating their involvement in data networks. They must also consider the differences in expectations, priorities, and values across stakeholder groups [38]. Empathy is essential for understanding participant hesitation and for eventually building trust, and a cultural shift may be warranted wherein data users (e.g. health professionals, researchers, and policymakers) consider policies on behalf of supportive and skeptical participants alike. If policy for expanding data networks is guided by the belief that there is an ethical imperative to share data in order to improve population health leading to support for data networking practices, data network governance should reflect this while also being responsive to other perspectives that may question this premise by ensuring benefits are delivered and respect for persons if not autonomy is preserved.

### Limitations

A probability-based sampling of the U.S. using companies such as GfK has limitations, since their participants are paid recurrent survey takers. While there was careful attention to the demographic composition and estimation of sampling weights to allow us to map from sample to U.S. population, we recognize we may not enough individuals in certain demographic categories (e.g. under-represented minorities across the education and income spectrum) to make strong, generalizable inferences. For example, it was surprising to us that the demographic variables were not significantly associated in this analysis, given the well-documented skepticism about the trustworthiness of the health system among minority populations [39,40]. However, we also recognize that this may not be an issue as the PEW foundation surveys and the American Academy of Sciences have recently reported that demographic factors are a variable and inconsistent predictor of skepticism about science and medicine in general, suggesting a need for future research into the relationship between demographic factors and willingness to participate in large health information networks [41,42,43,44].

Our findings were consistent with other surveys that show considerable mistrust and concerns about privacy in the general public [45], and with qualitative research that suggests concern about privacy and confidentiality in networked data sharing [46]. Surveys that limit data sharing to anonymized contexts show greater willingness to share, as do those of patients who are highly engaged with their care, such as those who participate in patient communities like PatientsLikeMe [47]. Our survey does not presume anonymity, given this condition cannot be guaranteed for the provision of care, quality improvement, and some applications in public and private research. It further broadens the perspective for the general public, going beyond other studies that address populations receiving care or identified in ambulatory clinic settings [48,49].

Our study points to factors in terms of the experience, values, and beliefs associated with a willingness to participate in multi-user data networks, but there is a need to further investigate additional attributes that might influence the public’s (patient and participant) views on data networks. For example, there may be differences in attitudes towards data networks depending on its purpose (e.g., for health care versus for research) [2] or the portal through which it is shared (e.g., through the Electronic Health Record or a quality improvement consortium or learning health system) [2]. There may be varying levels of trust contingent upon the institution accessing data and attitudes towards these institutions. We have chosen to investigate attitudes toward the health system in its most generalized form given that this is the level at which federal and state policy is made, and around which opinions and beliefs may be formed [29]. Future research might also consider whether participants with susceptibility to adverse health outcomes may perceive different vulnerabilities associated with data networks than the general public [5].

## Conclusion

Current policy for creating and expanding health data networks mirrors preferences of those most familiar with them and those with few concerns about harm, which does not reflect the attitudes of the general public. Future governance of data sharing networks, then, might consider two levers: 1) reducing data access to reflect the general public willingness to participate in data networks or 2) addressing concerns to ultimately improve the general public’s willingness to participate in data networks. Those making decisions about health information access should thus engage in dialogue with the public, including those skeptical of its benefits, to assess these options. Beyond informing policy for data sharing, such engagement is also likely to build trust and confidence in the types of large health information networks required for accelerated discovery and improved outcomes.

## Additional File

The additional file for this article can be found as follows:

10.5334/egems.288.s1 Supplementary tables. Variables contributing to indices used in this study (privacy, belief in medical conspiracy, confidence in governance, altruism, trust in providers).
