# Supplementary material for: Willingness to Participate in Health Information Networks with Diverse Data Use: Evaluating Public Perspectives
Source: EGEMS (Wash DC). 2019 Jul 25;7(1):33. doi: 10.5334/egems.288 (PMC6659576; doi:10.5334/egems.288)
Supplement: Supplementary tables. — Variables contributing to indices used in this study (privacy, belief in medical conspiracy, confidence in governance, altruism, trust in providers). [file egems-7-1-288-s1.pdf]

Supplementary tables

Indices

These indices were used in previous studies, e.g., (1, 2) to capture broad concepts like privacy, altruism, etc., that are best measured through multiple survey questions. In creating them, we performed Principal Component Analysis (PCA) on a larger a larger set of questions, using the PCA to maximize the variance explained in the multivariate distribution. Selecting a parsimonious set of questions meant that highly correlated variables would not unduly undermine the validity of the index.

Privacy index

For you, how true are the following statements	**Not at all true (%)**	**Somewhat true (%)**	**Fairly true (%)**	**Very true (%)**
I believe the privacy of my electronic personal health information is seriously threatened	**39.3**	**37.2**	**14.4**	**9.16**
I worry that private information about my health could be used against me	**38.5**	**35.9**	**14.4**	**11.3**
There are some things I would not tell my healthcare providers because I can't trust them with the information	**58.4**	**25.0**	**9.39**	**7.24**
Doctors could share embarrassing information about me with people who have no business having it	**38.2**	**37.0**	**13.2**	**11.5**
I worry my electronic health record is available to people who have no business knowing it	**30.2**	**38.4**	**16.5**	**14.9**
**Chronbach's alpha: 0.88**				

Belief in medical conspiracy index

For you, how true are the following statements	**Not at all true (%)**	**Somewhat true (%)**	**Fairly true (%)**	**Very true (%)**
The government |does not tell the public the truth about the dangers of vaccines	**42.2**	**33.7**	**14.5**	**9.62**
Some medical research projects are secretly designed to expose minority groups	**75.5**	**16.4**	**4.98**	**3.17**
The health care system experiments on patients without them knowing about the experiments	**57.1**	**31.5**	**7.92**	**3.51**
Health professionals don't tell you everything you need to know about medicines	**26.0**	**44.9**	**18.2**	**10.9**
I am concerned about serious adverse effects of vaccines	**50.7**	**29.8**	**11.0**	**8.60**
If I have a serious concern about my health, I can rely on the health system to address it	**12.2**	**35.5**	**36.7**	**15.6**
**Chronbach's alpha: 0.77**				

Confidence in current governance index

For you, how true are the following statements	**Not at all true (%)**	**Somewhat true (%)**	**Fairly true (%)**	**Very true (%)**
Access to electronic health information is adequately regulated	**21.3**	**47.3**	**24.2**	**7.24**
Electronic health information is sufficiently protected by current law and regulation	**22.0**	**42.2**	**27.3**	**8.60**
Health researchers are sufficiently accountable for conducting ethical research	**14.4**	**41,1**	**30.8**	**13.8**
Physicians are sufficiently accountable for conducting ethical research	**12.0**	**39.9**	**33.0**	**14.9**
I am confident in the standards for keeping personal health information confidential	**17.4**	**40.6**	**30.3**	**11.7**
**Chronbach's alpha: 0.89**				

Altruism Index

For you, how true are the following statements	**Not at all true (%)**	**Somewhat true (%)**	**Fairly true (%)**	**Very true (%)**
I always find was to help others less fortunate than me	**8.60**	**47.0**	**33.4**	**11.1**
The dignity and well-being of all should be the most important concern in any society	**6.22**	**22.5**	**31.3**	**39.9**
One of the problems of today's society is that people are often not kind enough to one another	**7.01**	**26.2**	**31.6**	**35.2**
All people who are unable to provide for their own needs should be helped by others	**10.4**	**40.8**	**27.4**	**21.4**
**Chronbach's alpha: 0.69**				

Trust in medical providers index

For you, how true are the following statements	**Not at all true (%)**	**Somewhat true (%)**	**Fairly true (%)**	**Very true (%)**
Health care providers care most about making money	**24.7**	**41.7**	**18.1**	**15.5**
Health care providers have a good track record of using information responsibly	**7.69**	**38.9**	**39.1**	**14.3**
I trust health care providers to use my health information responsibly	**7.69**	**30.2**	**39.8**	**22.3**
Health care providers tell you everything you need to know about the dangers of different treatments	**17.0**	**36.3**	**36.5**	**10.2**
Health care providers do not care about helping people like me	**70.0**	**22.7**	**4.98**	**2.26**
**Chronbach's alpha: 0.79**				

References

1. Platt J, Kardia S. Public trust in health information sharing: implications for biobanking and electronic health record systems. Journal of personalized medicine. 2015;5(1):3-21.
2. Platt JE, Jacobson PD, Kardia SL. Public Trust in Health Information Sharing: A measure of system trust. Health services research. 2018;53(2):824-45.
